# The relationship between genital self-image and sexual quality of life in obese women

**DOI:** 10.1590/1806-9282.20241006

**Published:** 2025-03-31

**Authors:** Özlem Süzer, Hamide Aygör

**Affiliations:** 1 Necmettin Erbakan University, Institute of Health Sciences – Konya, Turkey.; 2 Necmettin Erbakan University, Faculty of Nursing, Department of Obstetrics and Gynecology Nursing – Konya, Turkey.

**Keywords:** Body image, Obesity, Quality of life, Women

## Abstract

**OBJECTIVE:**

This study was carried out to evaluate the relationship between genital self-image and sexual quality of life in obese women.

**METHODS:**

This is a descriptive and correlational study. A personal information form, the Female Genital Self-Image Scale, and the Sexual Quality of Life—Female Questionnaire were used to collect the data.

**RESULTS:**

The mean total Genital Self-Image Scale score of the participants was 20.73±4.53, and their mean total Sexual Quality of Life Questionnaire score was 87.22±16.75. A strong positive relationship was determined between genital self-image and sexual quality of life.

**CONCLUSION:**

Healthcare professionals should evaluate the sexual quality of life of obese women, along with their genital self-image, so that appropriate sexual counseling and holistic care can be provided to them.

## INTRODUCTION

Body image involves the thoughts and feelings of an individual regarding their body^1^. "Genital self-image" is the aspect of body image that is associated with sexual satisfaction and functionality. Genital self-image is defined as the "thoughts, feelings, and attitudes of a person about their genitals^2,3^." Women who have a positive genital self-image feel more attractive. This is reflected positively in their sex lives and increases their sexual quality of life^4,5^. Increased body mass index (BMI) values were found to cause a negative genital self-image^6^. Taskın Yılmaz et al. also stated that obese women had a more negative body image and lower sexual quality of life, and a positive body image, raised sexual quality of life. In another study^7^, Carrilho et al. reported that there was no significant relationship between obesity and sexual functioning in obese women, and obesity was not an independent factor affecting the sex lives of these women^8^. In their systematic review and meta-analysis study, Firmino Murgel et al. revealed that hirsutism, BMI, and infertility could affect the emotions and sexuality of women diagnosed with polycystic ovary syndrome (PCOS)^9^.

Difficulties in the diagnosis and treatment of sexual dysfunctions are encountered due to several factors such as limited/insufficient time allocated to sexual health by healthcare workers during visits, the discomfort felt by both healthcare professionals and patients while discussing sexuality-related topics, the absence of a single etiological factor in sexual dysfunction cases, and the presence of multiple dysfunctions in one patient. The discomfort caused by sexual dysfunctions may influence the person's quality of life by causing a decrease in their self-image and self-esteem^10^. Although obesity is known as a risk factor for sexual dissatisfaction in men, the relationship between sexual satisfaction and body fat ratios in women has not been clarified yet^11^. The literature review conducted for this study revealed previous studies investigating BMI and genital self-image^6,12^, sexual quality of life in obese women^7,13^, sexual functioning and genital self-image in obese women^5,14,15^, and genital self-image and sexual quality of life in women^16^. However, no study investigating the relationship between genital self-image and sexual quality of life in obese women could be found in the relevant literature. To fill the gap in the literature on this topic, this study was carried out to examine the relationship between genital self-image and sexual quality of life in obese women.

## METHODS

### Design

This study was conducted with a descriptive and correlational design.

### Population and sample

The population of the study included women who presented to the Diet Clinic of a university hospital in the Central Anatolia Region of Turkey between December 21, 2023, and March 7, 2024. The sample size required to conduct the study was calculated using the G*Power 3.1.9.4 program based on the independent-samples t-test value^13^. With an effect size of 0.44, a 5% margin of error, a power of 90%, and a 95% confidence interval, the minimum required sample size was found to be 222. The study was completed with 222 participants.

The sample of the study included women at or over the age of 18 years, who were married, were at least secondary school graduates, were sexually active, did not have any cognitive, sensory, or verbal communication barrier, had BMI values equal to or greater than 30, and were visiting the Diet Clinic for the first time. The sample excluded pregnant women, menopausal women, and those who had any gynecological malignancies such as active breast, cervical, uterine, or ovarian cancer.

### Data collection instruments

Personal Information Form: The form consisted of a total of 14 questions, including sociodemographic and obstetric characteristics.

Female Genital Self-Image Scale (FGSIS): FGSIS was developed by Herbenick and Reece to evaluate the genital self-image of women^17^. The Turkish validity and reliability study of the scale was conducted by Ellibes Kaya et al. It is a 7-item, 4-point Likert-type scale. The minimum and maximum scores on the scale are 7 and 28. Higher scores are interpreted as a more positive genital self-image. Ellibes Kaya et al. reported the Cronbach's alpha internal consistency coefficient of the scale as 0.81^18^. In this study, this coefficient was found to be 0.82.

Sexual Quality of Life Questionnaire-Female (SQOL-F): SQOL-F was developed by Symonds et al. to assess the sexual quality of life of women^19^. The Turkish validity and reliability study of the scale was carried out by Tuğut and Gölbaşı^20^. SQOL-F is an 18-item, 6-point Likert-type scale. The respondent is asked to respond to the items based on her sex life in the last 4 weeks. The score range of the scale is 18–108, where higher total scores indicate better sexual quality of life. Tuğut and Gölbaşı reported the Cronbach's alpha internal consistency coefficient of the scale as 0.83^20^. In this study, this coefficient was found to be 0.90.

### Data analysis

The collected data were analyzed using the SPPS 25 (IBM Corp. Released 2017. IBM SPSS Statistics for Windows, Version 25.0. Armonk, NY: IBM Corp.) program. Descriptive statistics are presented as percentage, mean, standard deviation, median, minimum, and maximum values. The statistical analyses included independent-samples t-test, Mann-Whitney U test, one-way ANOVA, the Kruskal-Wallis H test, Pearson's correlation analysis, and multiple linear regression (backward method) analysis. The level of statistical significance was accepted as p<0.05.

### Ethical aspect of the study

Before starting the study, ethical approval was obtained from the Scientific Research Ethics Committee for Health Sciences at Necmettin Erbakan University (2023/567). Permission was then received from the institution where the study would be conducted (2023/E-14567952-900-425692). Potential participants were given information about the study prior to data collection, and those who agreed to participate provided verbal and written consent before they participated in the study.

## RESULTS

The participants had a mean age of 37.70±6.44 years. Most of the participants were secondary/high school graduates (43.7%), 81.1% were not working, and 89.2% were satisfied with their sex lives. The spouses of 46.4% of the participants were secondary/high school graduates, and the spouses of 93.2% were working.

The mean FGSIS score of the participants was 20.73±4.53, and their mean SQOL-F score was 87.22±16.75. The SQOL-F scores of the participants had a strong positive correlation with their FGSIS scores (p<0.01) and weak negative correlations with their age (p<0.05), the age of their spouse (p<0.01), and their duration of marriage (p<0.01).

The effects of 15 independent variables (age, education level, working status, monthly income, family type, age of spouse, education level of spouse, working status of spouse, marriage duration, history of miscarriage, history of abortion, number of children, last mode of delivery, satisfaction with sex life, and FGSIS scores) on the SQOL-F scores of the participants were tested using multiple linear regression analysis (backward method). Because the effects of the variables of age, education level, working status, monthly income, family type, working status of spouse, marriage duration, history of miscarriage, history of abortion, number of children, and last mode of delivery on the SQOL-F scores of the participants were insignificant, they were consecutively removed from the regression model (p>0.05). It was determined that the four variables remaining in the model (age of spouse, education level of spouse, satisfaction with sex life, and FGSIS scores) had significant effects on the SQOL-F scores of the participants. The descending order of significance of the effects of the four remaining variables according to their β coefficients was as follows: FGSIS scores (β=0.396, p<0.001), satisfaction with sex life (β=-0.325, p<0.001), age of spouse (β=-0.137, p<0.05), and education level of spouse (β=0.105, p<0.05). These four variables explained 40.7% of the total variance in the SQOL-F scores of the participants. A one-unit increase in FGSIS scores corresponded to a 0.396-unit increase in SQOL-F scores, a one-unit increase in satisfaction with sex life corresponded to a 0.325-unit increase in SQOL-F scores, a one-unit increase in the spouse's age corresponded to a 0.137-unit decrease in SQOL-F scores, and a one-unit increase in the spouse's education level corresponded to a 0.105-unit increase in SQOL-F scores (Table 1).

**Table 1:** Effects of independent variables on Sexual Quality of Life Questionnaire-Female scores: multiple linear regression analysis (n: 222).

Variables	B	SE	t	p	B	95%CI for B		Collinearity statistics	
						Lower	Upper	Tolerance VIF	
(Constant)	85.154	8.981	9.482	0.000		67.449	02.859		
Spouse age	-0.316	0.124	-2.554	0.011	-0.137	-0.560	-0.072	0.959	1.042
Spouse education level	2.518	1.314	1.916	0.047	0.105	0.073	5.109	0.920	1.087
Satisfaction with sex life	17.350	3.092	5.611	0.000	0.325	23.445	11.254	0.824	1.213
FGSIS	1.483	0.218	6.815	0.000	0.396	1,054	1.912	0.820	1.219

R: 0.651; R²: 0.424; Adjusted R²: 0.407; F: 25.474; p=0.001; Durbin-Watson: 1.894. VIF, variance inflation factor; B, nonstandardized beta; β, standardized beta; t, significance of beta; p, significance; SE, standard error; CI: confidence interval; FGSIS: Female Genital Self-Image Scale.

## DISCUSSION

In societies shaped based on gender, social expectations from men and women are different, and these expectations are usually more serious for women. This may lead women to pay more attention to their bodies compared with men and feel more under pressure regarding their physical appearance and beauty. Especially the views and expressions of the sexual partners of women are highly influential on their genital self-image^6^.

In this study, 89.2% of the participants stated that they were "satisfied" with their sex lives. Küçük et al., similarly, reported that the sexual satisfaction levels of obese women were "high."^11^ In another study, 43.7% of obese women described their sex lives as good/very good^13^. According to Silva et al., 52.1% of women with PCOS were of normal weight, 33.3% were overweight, and 14.6% were obese, while 81.3% were satisfied with their sex lives^21^. In the literature, it was emphasized that while obesity was a risk factor for sexual dissatisfaction in men, the relationship between sexual satisfaction and body fat content in women was still unclear^11^. Differences in the results of different studies may be attributed to cultural factors. In Turkish society, being heavier represents the fertility and vitality of women. While assessing potential brides, mothers-in-law consider thin women unhealthy and weak. This positive view on being overweight may have been reflected positively in the sex lives of women by increasing their self-esteem.

A "strong" positive relationship was identified between the SQOL-F and FGSIS scores of the participants of this study. Likewise, Taskın Yılmaz et al. showed a positive relationship between body image and sexual quality of life in normal-weight, overweight, and obese women^7^. Küçük et al. found a relationship between body image and self-esteem in obese women and stated that as body image and self-esteem improved, sexual satisfaction increased^11^. In other studies that were conducted with women, similarly, "weak" positive relationships were identified between genital self-image and sexual quality of life^16^ and genital self-image and sexual functioning^14^. The feelings of women about their genitals were associated with their positive and negative feelings about sexuality^2^. The positive feelings, thoughts, and perceptions of the participants regarding their genital area may have affected their sexual quality of life positively by leading them to feel more attractive and confident in front of their partners.

It was found in this study that age, spousal education level, satisfaction with one's sex life, and FGSIS scores affected the SQOL-F scores of the participants significantly. These four variables explained 40.7% of the total variance in SQOL-F scores. As a similar result, Taskın Yılmaz et al. found that as the satisfaction of normal-weight, overweight, and obese women with their body image increased, their sexual quality of life also increased. In their study, body image was determined to explain approximately 15% of the total variance in sexual quality of life, and a one-unit increase in sexual quality of life corresponded to a 0.38-unit increase in body image^7^. Küçük et al. reported that body image and self-esteem affected sexual satisfaction in obese women^11^. In yet another study carried out with women, genital self-image was discovered to influence sexual functioning^22^. Yaşar Ayan and Beydağ demonstrated that sexual quality of life was affected by being satisfied with one's sex life, being compatible with one's sexual partner, and thinking that the opinion of one's sexual partner about one's genitals. A one-unit increase in satisfaction with sex life corresponded to a 0.178-unit increase in the sexual quality of life scores of women^16^. The results of this study were in agreement with those in the literature.

## CONCLUSION

A strong positive relationship was determined between the genital self-image levels and sexual quality of life levels of obese women. The age of the woman, the education level of her spouse, her satisfaction with her sex life, and her genital self-image explained 40.7% of the total variance in her sexual quality of life.

Healthcare professionals should evaluate the sexual quality of life of women along with their genital self-image. Considering that the education levels of partners are a significant predictor of sexual quality of life, in the field of sexual health counseling, it is important to encourage the participation of partners in counseling sessions. Furthermore, considering that age and monthly income are predictors of sexual quality of life, older men and women and those who have lower socioeconomic status should be given priority in counseling.

### Limitations

The results of this study are limited to the sample in which the study was conducted and may not be generalized to the entire society. Including only obese women presenting to one diet clinic was a limitation.

## Data Availability

The dataset is available from the corresponding author on reasonable request.
